# Post-pandemic transformations: How and why COVID-19 requires us to rethink development

**DOI:** 10.1016/j.worlddev.2020.105233

**Published:** 2021-02

**Authors:** Melissa Leach, Hayley MacGregor, Ian Scoones, Annie Wilkinson

**Affiliations:** Institute of Development Studies at the University of Sussex, UK

**Keywords:** Epidemics, Development, Inequality, Uncertainty, Transformation, COVID-19

## Abstract

COVID-19 is proving to be the long awaited ‘big one’: a pandemic capable of bringing societies and economies to their knees. There is an urgent need to examine how COVID-19 – as a health and development crisis - unfolded the way it did it and to consider possibilities for post-pandemic transformations and for rethinking development more broadly. Drawing on over a decade of research on epidemics, we argue that the origins, unfolding and effects of the COVID-19 pandemic require analysis that addresses both structural political-economic conditions alongside far less ordered, ‘unruly’ processes reflecting complexity, uncertainty, contingency and context-specificity. This structural-unruly duality in the conditions and processes of pandemic emergence, progression and impact provides a lens to view three key challenge areas. The first is how scientific advice and evidence are used in policy, when conditions are rigidly ‘locked in’ to established power relations and yet so uncertain. Second is how economies function, with the COVID-19 crisis having revealed the limits of a conventional model of economic growth. The third concerns how new forms of politics can become the basis of reshaped citizen-state relations in confronting a pandemic, such as those around mutual solidarity and care. COVID-19 demonstrates that we face an uncertain future, where anticipation of and resilience to major shocks must become the core problematic of development studies and practice. Where mainstream approaches to development have been top down, rigid and orientated towards narrowly-defined economic goals, post-COVID-19 development must have a radically transformative, egalitarian and inclusive knowledge and politics at its core.

## Highlights

• COVID-19 has exposed major faultlines and fragilities in current systems.
• A structural-unruly duality characterises emergence, progression and impact.
• Mainstream development thinking and practice are part of the problem.
• Transformations are needed in science-policy, economies and governance.
• Resilience, equity, diversity, care and inclusive politics are required.

## 1 Introduction

The COVID-19 pandemic has sent shock waves through societies and economies around the world. The impacts of the disease and of measures to control it have raised questions about epidemic preparedness and more generally about development, past, present and future. What made the world so vulnerable? Why were individual countries and the international community not better prepared? And what needs to change to mitigate harm from future threats, particularly with respect to the most politically, economically, socially and clinically vulnerable? Epidemics often provoke such reckoning, but rarely on this scale. The massive global health and development crisis enwrapped with the COVID-19 pandemic has exposed the limits of conventional framings of development both North and South. Drawing on our experience of working on past major disease outbreaks, and of studying social change and transformation, this article focuses on questions of, what next? While the pandemic has exposed fractures and contradictions in conventional ways of acting – most notably mainstream approaches to capitalist development – it is also suggesting new ways forward.

Existing fragilities in systems of all kinds – be they those that assure health and wellbeing, food, sustainable livelihoods, resilient ecologies, resource access, employment, trade, finance, inclusive governance, citizen rights and more – have been highlighted, and sometimes intensified, by COVID-19. At the time of writing, impacts are still unfolding fast and remain uncertain. It is already clear, however, that they are being felt unevenly, exposing differences of vulnerability across geographies and social groups. Further outcomes seem inevitable: the deepening of poverty; increases in multiple, intersecting inequalities; a worsening of chronic fragility and instability and potentially intensified authoritarianism.

‘Development’ – understood as progressive social, economic and political change – is rapidly being undone as COVID-19 threatens collective futures. Long-dominant development models, such as those promoting economic growth, market liberalisation, globalisation, carbon-intensive industries and command-and-control planning regimes, are now under unprecedented challenge. Mainstream approaches to pandemic preparedness and response – encapsulated in the World Health Organisation’s guidelines for health emergency preparedness (WHO, 2017) – reproduce central features of these dominant development approaches, and have emphasised globally-standardised, top-down control measures, directed vertically at prioritising the disease outbreak. These approaches are now being rolled out for COVID-19, but their limitations are exposed as they clash with the complexity of varied social, economic and political settings and catalyse wider indirect impacts.

At the same time, often *ad hoc* and contingent alternatives for disease control and impact mitigation are being generated, rooted in local networks and solidarities. Potentials for strengthened global solidarities, interconnections and mutual (and reversed) learning are also emerging, challenging the deeply-rooted and problematic North-South boundaries and hierarchies that have pervaded so much development thinking and practice. These are all elements in growing calls to rethink aspects of both pandemic preparedness and response and development more broadly (e.g. Oldekop et al., 2020, Lambert et al., 2020), towards positive post-pandemic transformations that envision radically different futures.

Moving beyond the plethora of fragmented opinion pieces and commentaries on these issues sparked by COVID-19, this article offers a more consolidated, theoretically-grounded contribution to what we term post-pandemic transformations, considering the implications for development thinking and practice. We argue that the origins, unfolding and effects of the COVID-19 pandemic require analysis that addresses both structural political-economic conditions alongside far less ordered, ‘unruly’ processes reflecting complexity, uncertainty, contingency and context-specificity. This synthesis of two strands of analysis applies to ecological, social, economic and political systems and processes and to the interactions between them. While these two strands reflect deeper social theories and analytical traditions that can be in tension in considerations of radical transformations (cf. Scoones et al., 2020), we argue that bringing them together is essential in this moment of ‘rethinking’. Too often in studies of development and change, including around global health issues, analyses of structural conditions – of power, politics and economic relations – and understandings of local agency and mobilisation – with all the attendant complexities, contingencies and uncertainties of particular contexts, histories and ecologies – have been pursued in separate disciplinary siloes, or have been side-lined altogether with a focus simply on instrumental policy interventions.

In this article, we develop our argument through two parts, each exploring themes that the COVID-19 pandemic has exposed, but grounding the discussion in past work on disease outbreaks where analyses have productively used both ‘structural’ and ‘unruly’ approaches. The first part of the article explores pandemic origins, unfolding and impacts. We show how outbreak origins, especially when disease agents can transfer from animals to humans and cause infection, require analysis in terms of both structural political economies and ecologies and of people’s interactions with unruly, non-equilibrium, non-human natures. Whether and how outbreaks become epidemics and pandemics, who they affect and how, similarly requires attention to structural inequalities and vulnerabilities and forms of structural violence in health systems and health inequalities and wider society, as well as to diverse, more contingent, complex and context-specific processes and experiences.

This structural-unruly duality in the conditions and processes of pandemic emergence, progression and impact in turn inflects how post-pandemic transformations are thought about and enacted. In particular, three challenge areas for rethinking are revealed, rooted in themes surfaced by COVID-19, which we explore in the second part of the article. The first is how scientific advice and evidence are used in policy, when conditions are on the one hand rigidly ‘locked in’ to established power relations and political-economic regimes and yet, on the other hand, so uncertain, reflecting unruliness in social-political-ecological processes. Second is how economies function, with the COVID-19 crisis having revealed the limits of a conventional model of economic growth and the wider capitalist structures it is embedded in, but also the need for plurality and negotiation around workable post-capitalist alternatives. The third concerns how new forms of politics can become the basis of reshaped citizen-state relations in confronting a pandemic – or indeed other crises such as climate change – with attention again needed both to structural transformations and to emergent, enabling practices, such as those around mutual solidarity and care.

Our analysis is informed by several broader bodies of literature that also, in different ways, address the relationships between structural conditions and specific, contingent, often unruly, contexts. For example, we are influenced by science and technology studies, and ideas about the role of expertise in policy and the importance of risk and uncertainty in framing decisions (Jasanoff, 2004, Wynne, 1992); by studies of ‘reliability management’ and the practices of professionals and functioning of administrative systems in critical infrastructures (Roe and Schulman, 2008); by political ecology, and how human-environment relations are influenced by politics and *vice versa* (Perreault et al., 2015); by feminist approaches, with their emphasis on unruly politics, co-operation, networks, social reproduction, care and humility (Harcourt and Nelson, 2015, Fraser, 1989) and by perspectives on alternative economies, ‘degrowth’ and the politics of green and just transformations (Newell, 2015, D’Alisa et al., 2014). None of these perspectives currently provide the mainstream foundations of development studies, although they increasingly appear at the margins. We believe this must change. COVID-19 should be a reminder that we face an uncertain future, where anticipation of, resilience to and rebuilding from shocks in a highly unequal world will be the core problematic of development studies and practice.

The starting point for our analysis in each part of the article is the COVID-19 pandemic, but we reinforce this with reflections from other disease outbreaks that we have studied in over a decade of research on the social dynamics of epidemics (Dry and Leach, 2010)^2^ and the politics of epidemic preparedness and response. These include the HIV/AIDS pandemic from the late 1980s (MacGregor, 2010); the avian and swine influenza outbreaks from the late 1990s and through the 2000s (Forster, 2012, Scoones, 2010); the West African Ebola epidemics from 2013 to 16 (Wilkinson and Leach, 2015) and Zika outbreaks in Latin America (Bachtold, 2020), alongside a range of zoonoses that have impacts on local livelihoods(Bardosh, 2016, Cunningham et al., 2017). No epidemic or pandemic outbreak is the same, but there are some striking similarities across these experiences and with COVID-19.

Lessons have frequently been drawn for how to improve preparedness and response to (re)emerging infectious diseases, yet these have rarely been applied. Too often, there has been a narrow, technocratic response focused on medical innovation and public health surveillance, often framed by a securitisation discourse, as exercises in anticipating risk (Lakoff, 2017) or as governance failures that could be resolved by strengthened rules. Such shortcomings are mirrored in development approaches more widely. New outbreaks have repeatedly shown how the challenges of epidemics are not limited to health but are far broader, and often intrinsically political. Indeed, often missing as well are the connections between epidemic emergencies and longer-term development. Along with many others, our work has shown how important social science is to illuminate disease dynamics, their wider social and political contexts and more fundamental development implications. In the sections that follow we therefore link recent experiences with COVID-19 with longer-term debates around outbreaks, epidemics and pandemics, and suggest how these can help us rethink – and potentially catalyse transformation in - development more broadly.

## 2 Part 1: Pandemic origins and impacts

From its origins in late 2019 to now, the COVID-19 pandemic has wreaked havoc around the world. Understanding how it started, its unfolding in different regions and the very uneven ways in which people and places have been affected, requires us to attend both to structural drivers and conditions and to far more dynamic, more unruly and therefore more uncertain processes in the ways people have responded and how they interact with non-human natures and with each other.

### 2.1 Political ecologies and unruly socio-natures

The majority of new human diseases have come from wild animals, often via domesticated livestock or poultry (Jones et al., 2008). COVID-19 is one of these, along with HIV, H5N1, H1N1, Nipah, Ebola Virus Disease and others; and there will definitely be more. Zoonotic spillover events, where disease agents transfer between animals and to humans, occur frequently, but do not usually result in major disease outbreaks. It is only under certain circumstances, such as with SARS-CoV-2, where transmission between humans is easy, and mortalities low enough to allow significant viral spread but high enough to be a concern, that a new disease gets noticed. It is dramatic outbreaks affecting populations, particularly in rich, Northern countries, that tend to drive global health policies and attention.

But for considerable periods before the alarm is raised, viruses and other pathogens are circulating both in the wild and, in some cases, in human populations. A high fever and early death may be put down to malaria, for example, rather than a novel virus, and so emerging outbreaks go unnoticed, especially in contexts where diagnostic resources are limited. The reality is that in many countries people frequently become sick and die from unknown causes, but neither these deaths nor their causes are counted. HIV, for example, may have emerged through transfer from primates in Africa, but only gained significant public attention when AIDS struck populations in the US in the 1980s. We simply do not know how long SARS-CoV-2 was present in China before it was recognised as a novel, disease-causing virus.

Understanding disease emergence and spillover is therefore crucial if a preventive approach to novel zoonotic diseases is to be promoted. However, our understanding of such processes is frequently limited, as the environments from where diseases emerge are unruly, complex and uncertain. There are various approaches to tracing the origins of disease including phylogenetics and ecological sampling, but the science is inexact. Here again, uncertainty prevails. There are plenty of ‘just-so’ stories that point fingers of blame to particular sources, but these are largely tentative, and often misleading. For example, in the case of COVID-19, the so-called ‘wet markets’ of urban China have been highlighted as the most likely source of disease risk, while in the avian influenza outbreak it was small-scale poultry farmers. The public health response is frequently technocratic – to close down markets or exterminate poultry, for example – often without deeper knowledge of origins and transmission pathways or local conditions and regulatory systems (Lynteris and Fearnley, 2020). The results can be both ineffective in public health terms and damaging to the lives and livelihoods of small-scale farmers and traders.

Understanding and responding to zoonotic disease must recognise both the complex dynamics through which humans, animals and unruly ecologies interrelate, and the structural political-economic conditions shaping the likelihood of spillover and influencing whether a spillover event becomes an epidemic. As the structural relations of environments, human habitation and production systems are transformed, dynamic disease ecologies create new patterns of vulnerability. Intensive livestock production increases the probability of outbreaks of high-impact animal diseases, through confinement of large numbers of animals with low genetic diversity in small spaces, and with high turnover; all conditions that can enhance pathogen virulence. Vulnerabilities can also arise from increased human-wildlife interaction, exacerbated by habitat destruction due to commercial agriculture, unchecked urbanisation and land and resource grabs. Pathogen spread can be facilitated by intensified interactions between disease-hosting wildlife and farmed, traded or domestic animals who act as intermediate hosts in transmission to humans. These dynamics are in turn shaped, and sometimes amplified, by wider human-ecosystem changes and their structural causes, whether climate change, biodiversity loss, land and forest change, settlement patterns, population displacements due to conflict or new markets and investments in remote rural areas. The ‘efficiencies’ of global trade have also, in many circumstances, paved the way for increasingly uniform farming systems and impoverished landscapes without the disease ‘firebreaks’ of biodiversity (WHO/CBD, 2015).

Structural drivers and the complex dynamics of socio-ecological change thus have to be considered in tandem if we are to beware over-simplistic, linear, causal narratives of zoonotic events; very often diseases emerge not through a single event – as in the spread from a ‘patient zero’ constructed in the epidemiological imagination – but through a wider set of intersecting, structural processes over broad temporal and spatial scales, with the interaction of politics, social relations and ecology becoming central (Wallace, 2016). For example, while zoonotic disease spillover likelihood is increased in farmers’ markets that contain multiple wild animals in close proximity and unsanitary conditions, we must ask how such conditions emerge: what are the politics of regulation in such places; who makes use of such markets; how are animals hunted and captured, for whom, and so on? It may be that the ‘cause’ is not the ‘wet market’ itself but the wider ecological changes, including declines in biodiversity in the areas animals come from, with structural economic drivers pushing hunters and trappers to go further to gain access to resources, as land is expropriated for other uses (Wallace et al., 2020).

In the outbreaks of avian and swine influenza it was the changing political ecologies and economies of poultry and pig farming that were key. For avian influenza, it was the growth of medium-scale, industrial units with limited biosecurity in fast-growing southeast Asian nations with growing demand for poultry meat that was central to outbreak dynamics (Scoones, 2010). Meanwhile, for swine flu, it was the industrial production of pork, across the southern US and Mexico, led by some large, well-connected agribusiness firms, which provided the conditions for the outbreak (Forster, 2012). In both cases, changing agricultural production and food systems, and the circuits of capital involved, intersected with particular context-specific farming practices and human-animal relations. As humans transform environments, disease outbreaks are of course inevitable, but always uncertain. Thus, after avian influenza, the ‘big one’ (i.e., a global outbreak with massive consequences) was expected to come from poultry in Asia, facilitated by circulation through wild bird populations; but, just a few years later, the next pandemic came from pigs in the Americas. And then COVID-19, which has turned out in many respects to be the long-expected major global pandemic, came from a coronavirus and a different set of, as-yet-unknown, animal intermediaries.

Meanwhile, no-one predicted the Zika virus outbreak in South America or the major West African Ebola epidemic from 2013. Ebola is presumed to be a virus hosted by bats, and in some accounts the West African outbreak resulted from deforestation and habitat fragmentation increasing human-bat contact (Bausch and Schwarz, 2014). Yet, whilst forest ecosystem changes and their structural causes – in commercial agriculture, logging and settlement expansion – are significant processes, linear deforestation and increased human-bat contact are unsupported in this part of West Africa (Fairhead and Leach, 1996). Instead, this outbreak – and earlier, smaller ones in East and Central Africa – reflects a more complex, unruly mix of interactions between bats, intermediate wildlife hosts, dynamic forest ecologies and human-to-human transmission. These intersect with social and livelihood dynamics (e.g. migration, mining and hunting) unfolding over wider temporal and spatial scales (Fairhead and Millimouno, 2017). That this particular outbreak spread into a multi-country epidemic with (conservative estimates of) over 28,000 cases and 11,000 deaths in turn reflects further structural conditions – including fragile and distrusting state-citizen relations, impoverished health systems, rapid urbanisation and the legacies of conflict (Wilkinson and Leach, 2015).

This picture of structural social and political-ecological conditions interplaying with fast-changing, non-linear local human-animal-ecological dynamics to generate outbreaks, uncertain in their timing and precise forms, is repeated in our further studies of zoonotic disease dynamics in African settings including Lassa Fever, Rift Valley Fever, Nipah and trypanosomiasis (Leach et al., 2017). In Zimbabwe, for example, changing patterns of transmission of trypanosomiasis (sleeping sickness) to people reflects the spread of agriculture that has confined forest (and the tsetse flies that transmit the disease) to small patches beside rivers and in valleys and the shifting patterns of land-use and livelihoods that affect use of these landscapes. In Kenya, Rift Valley Fever, affecting livestock, takes both endemic and outbreak forms, and disease spread has been enhanced by the expansion of habitats for mosquito vectors through irrigation for commercial agriculture, interacting with uncertain weather events.

In sum, as COVID-19 as well as past disease outbreaks have repeatedly shown, the underlying causes and consequences of ecological disruptions reflect structural social and political-economic conditions. This is of course long-recognised by approaches in political ecology (Perreault et al., 2015), yet it is striking how many development policy approaches, including in the context of climate change, are reduced to treating symptoms, focusing on the behaviours of those in closest interaction with animals, or addressing problems as if they were one-off disasters. As insights from ‘non-equilibrium ecology’ more generally have shown, simplistic, deterministic approaches to planning and intervention in development quickly unravel (Scoones, 1999). A wider perspective that accepts the unruliness of natures and the non-linearity of outcomes suggests a different approach; one that is more respectful, contingent and precautionary. This unruliness of diverse socio-natures in turn requires a greater attention to dynamic ecologies, alongside attention to the structural political and economic underpinnings and consequences, across development research and practice.

### 2.2 Structural vulnerabilities and dynamic inequalities

Epidemics are often said to be mirrors to society and COVID-19 has revealed a highly unequal world. It has highlighted inequalities and structural vulnerabilities, often the result of long histories of marginalisation. Although the virus initially spread in richer countries, the trends soon reversed. More affluent groups, with jobs that could be done at home and did not involve exposure to the virus, were more able to comply with control measures thus keeping them safer; they were also less vulnerable to severe disease and death due to socio-economic gradients of underlying health conditions (Patel and Hardy, 2020). In the UK and the US black and minority ethnic groups died at higher rates than others (Freshour and Williams, 2020). The racial, gendered and class dimensions of disease are vividly shown. This is what Farmer (2001) refers to as ‘structural violence’, where diseases disproportionately affect the poor and marginalised. The magnitude and impacts of the 2013–16 Ebola epidemic in West Africa have also been analysed in terms of structural violence, here reflecting deeply-rooted histories of slavery, resource dispossession, state and foreign investment and under-developed health systems compromised by ‘structural adjustment’ (Leach, 2015, Wilkinson and Leach, 2015). Farmer (2014) points to the resource shortages in weak health systems and the forces that had driven underinvestment and inequalities in access to healthcare in West Africa as the underlying causes of Ebola mortality. These factors thus could be seen to constitute further forms of structural violence.

We have explored the nuances of the social dimensions of health in our studies of zoonotic diseases in Africa, where we asked, ‘who gets sick and why?’ (Dzingirai, Bett et al., 2017). In the case of trypanosomiasis in Zimbabwe it was those who herded domestic animals or went hunting or collected wild fruits who were exposed to the tsetse flies in areas where wild animals were also present, and so most likely to contract the disease. In the case of Lassa fever in Sierra Leone, women were more at risk due to time spent in the houses, kitchen gardens and dry season horticulture patches in which *Mastomys* rodents, the animal hosts of Lassa virus, concentrated. Across our studies, it was a mix of occupation, gender, age and wealth that were the major factors resulting in an increase in exposure and so likelihood of contracting a disease (Leach et al., 2017). Ecological disease incidence maps and population-level correlations tell only part of the story. It is the social and political factors, rooted in unequal patterns of vulnerability, often linked to deep structural factors – such as colonial settlement, migration circuits, external investments and histories of war and conflict – that are the major determinants (Dzingirai, Bukachi et al., 2017).

A similar story of differential disease risk is demonstrated in the case of Zika in Latin America, where it was amongst those living in poor neighbourhoods with poor sanitation, inadequate drainage and high mosquito populations that the disease was most common. Women in dense, low-income urban settlements were more likely to give birth to babies with Zika Congenital Syndrome, but they were also less likely to have access to safe abortion and other sexual and reproductive health services such as contraception. The impact of having a child with disability was more acute for this group of women, many of whom have had to give up employment to take on care responsibilities. Even though Brazil has systems for free healthcare and social protection provision, a long history of inequality, engendered by persistent structural violence, makes it more difficult for women in marginalised situations to gain access to support (Bachtold, 2020).

Inequalities are revealed not only in relation to disease burdens, but also in how people are affected by disease control efforts. While there are understandably heightened fears about the potential for uncontrolled COVID-19 transmission in ‘slums’ and informal settlements, it is the control measures that have significantly affected residents and the informal economies upon which they depend. These livelihoods and people have been systematically undervalued, undercounted and thus rendered invisible, and assumed to be marginal. Policies have not factored in questions about the feasibility of control measures or their impacts. Health messages to ‘socially-distance’, to wash hands regularly with running water and soap and to stay at home are less achievable in poor, crowded informal settlements with limited water and sanitation infrastructure, and where people must work every day to survive – the choice is between hunger or (potential) disease (Wilkinson, 2020, Hrynick et al., 2020 Backspace).

In the SARS outbreak in China, migrant labourers were at risk of disease because of their tenuous status and limited access to healthcare, but measures to stem the epidemic that targeted them as sources of infection had the unintended consequence of further mobility and disease spread (Xiang, 2003). Similarly, the COVID-19 lockdown of businesses in metropolitan centres in India has resulted in the mass movement of labour migrants back to their rural villages, often over long distances, again spreading infection. Across the world, violence has been used to enforce impossible restrictions on people. Although some countries have attempted to mitigate this with food packages or social protection schemes, these have rarely been sufficient. This has exposed the artificial divides between informal and formal contexts and economies, showing how informal settlements and informal economies are not peripheral to the cities (and global economies). Rather, they provide essential services such as waste collection, domestic work and manufacturing, at subsidised rates based on their informal status for which the poor pay the ultimate price.

State-led responses to a disease outbreak therefore often replicate biases within development, reinforcing alienation, marginalisation and stigmatisation. The simple instruction to ‘stay at home’ overlooks the grim reality that many people do not have safe or stable homes. There has been an increase in domestic abuse following quarantines and lockdowns. Equally, heightened discrimination and vulnerabilities of those living with disabilities, addictions or chronic conditions is witness to the uneven consequences of standardised public health measures on diverse populations (Wilkinson, 2020, Hrynick et al., 2020). Labour migrants, mobile pastoralists, informal traders, sex workers and people whose daily provisioning depends on multiple, overlapping living and household arrangements are hugely affected by top-down measures that do not take account of diverse livelihoods and lifestyles, with different notions of ‘home’ and different requirements of movement. This is reflective of the blindness to inequality and social difference of much technocratic development, well beyond edicts on pandemic health measures.

Thus, contrary to the standard ‘we are all in this together’ narrative, diseases and public health responses to them clearly discriminate, accentuating long-standing structural inequalities locally, nationally and globally, as well as interplaying with multiple, dynamic, negotiated sources of marginalisation such as those that have emerged as the epidemic unfolds and have required contingent responses. Attention to inequality – and the effects of uneven development – has returned to development thinking and policy in recent years, and COVID-19 highlights why this must become even more central. The world is becoming a more unequal place economically, both globally (Milanovic, 2016) and within countries (Picketty, 2014). Multiple dimensions of inequality beyond the economic also require more attention, acknowledging the ways that social, gender, economic, political, cultural, spatial, environmental and knowledge inequalities interact and intersect (ISSC, IDS and UNESCO, 2016). COVID-19 has also highlighted the dynamic nature of such intersections as old vulnerabilities are made more apparent and new ones have emerged.

## 3 Part 2: Towards post-pandemic transformations

In answering how COVID-19 – alongside other diseases – prompts reconsideration of our conceptions of development we have highlighted two themes – unruly natures and socio-political processes, and deepening structural inequalities. The question now arises: what are the implications for post-pandemic transformations, and for rethinking development?

In this second part of the article, we now go on to explore how challenges arise for thinking and action in three key areas – for science and decision-making, for building resilient economies, and for citizen-state relations. For each area, we consider, again, what has been learned during the COVID-19 pandemic alongside other epidemics, and we discuss implications for addressing future health and other crises, and for development more generally.

### 3.1 Rethinking science, policy and uncertainty

How should scientific advice and evidence be used in policy for disease outbreaks and indeed wider development issues? Here we confront the challenge that, on the one hand, the conditions of science advice and the science-policy processes in which they are embedded reflect deeply-rooted politics and institutional structures, yet advice must also respond to pervasive uncertainties and unfolding, dynamic complexities.

This tension certainly been the case for COVID-19. The global response has been heavily informed by epidemiological models, now a mainstay of outbreak responses. There are many competing versions, all using different data sources. Not surprisingly, this has led to many different predictions of what will happen, and what should be done (Rhodes et al., 2020). At one level, this is a healthy situation, with multiple scientists working in good faith to try and find a solution in the face of deep uncertainty; for example about transmission routes, co-morbidities, mortality rates, exposure levels, immune responses and age effects. The danger comes when such uncertainties, or indeed forms of ignorance, are obscured in the process, and alternative forms of knowledge and insight on the disease do not enter the conversation. Necessarily, models simplify the world, boiling it down to a few key estimates and parameters. Models themselves are therefore only as good as the assumptions informing them, and diverse perspectives – including from social science – can provide essential qualifiers and complements. Problems arise when these assumptions are not acknowledged, examined or adjusted. Perhaps even more than earlier epidemics, narrow epidemiological modelling expertise has been the dominant source of science advice during the COVID-19 episode (Enserink and Kupferschmidt, 2020). As politicians and advisers grapple with competing views and public pressure, the quantitative framing and false certainties of a model are appealing, and caveats are easily pushed aside. As the COVID-19 experience has shown, this can be dangerous, with lethal consequences (Sample, 2020).

During the avian influenza outbreaks in Southeast Asia, similar epidemiological models were used to predict spread, and guide intervention responses. In 2005 two models were published in *Nature* and *Science* (Ferguson et al., 2005, Longini et al., (2005)’) that focused attention on ‘at source’ control to avoid further human spread. The result was support for massive intervention to exterminate poultry (and wild duck and geese) populations across the region. At the same time, models of potential human–human transmission predicted huge numbers of potential mortalities (Scoones, 2010). Similar predictions were offered at the start of the 2013–16 Ebola outbreak in West Africa, with a Centers for Disease Control model predicting up to 1.4 million deaths (Meltzer et al., 2014). While helping to mobilise resources for international organisations, such predictions were based on major assumptions about a lack of human behaviour change and community response, which thankfully turned out not to be true (Richards, 2016).

What was missing during the avian influenza outbreaks was a sense of conditions on the ground and the priorities of local people and therefore what this meant for how control measures would play out. Veterinarians complained that mass extermination of backyard chickens would cause huge damage to livelihoods and economies and would have little impact on spread, even among birds. Other foci for disease outbreaks were likely much more the cause of spread, notably commercialised medium-scale poultry production units with limited biosecurity. Meanwhile, medical doctors who had dealt with patients both in Hong Kong in 1997 and across southeast Asia from 2004 were raising questions about transmission rates through case reports. There was no argument for complacency, but other insights – whether from field veterinarians or hospital doctors and nurses – were important, yet largely overlooked. It turned out that transmission of the avian H5N1 virus was limited between humans, and only around 350 human deaths were recorded globally, while over a billion chickens were destroyed and many livelihoods were affected.

Likewise, local social conditions and responses were ignored in the initial modelling of Ebola in West Africa, with associated policies and investment focusing heavily on providing large numbers of Ebola treatment beds. The linear assumptions about disease transmission did not align with the region’s embedded, unruly entanglements of kinship, travel and trade. Local social and cultural practices around funerals, care and visiting proved central to the course of the epidemic and, as villagers and front-line workers co-developed understandings of infection, they adapted their practices to balance social and disease issues accordingly – for instance through creating non-physical burial rituals and locally-led quarantine practices that suited social and livelihood contexts (Richards, 2016). Evidence from local learning and action gradually fed into policy responses, facilitated by networks of social scientists, enabling approaches to become more community-engaged, sensitive and effective. By the time many of the originally-planned treatment units were completed, the epidemic had already subsided, largely due to community-led behaviour change.

This is not an argument against the use of epidemiological models, but one for a recognition that they have social and political lives (Leach and Scoones, 2013). The uncertainties embedded in simple models are legion, and evidence emerging from modelling exercises must be triangulated with other sources. Local knowledge of how diseases spread, what the impacts are and how they can be managed in context-appropriate ways is crucial (Scoones et al., 2017). In the COVID-19 case, multiple pitfalls of modelling have emerged (Sample, 2020). In the UK, it was only once the modellers based at Imperial College London (using the same core model as used for avian influenza in the 2000s) added in data from hospitalisation in Italy, rather than using estimates from past seasonal influenza outbreaks, that the advice changed. The driving concern was that National Health Service critical care capacity could be exceeded. Yet, doctors and patients in China had plenty of experience in the weeks and months before, and this clearly indicated the effects of this novel virus were distinct from influenza and that radical containment to suppress transmission was necessary if health systems were not to be overwhelmed (Horton, 2020).

Outside times of crisis, as in disease outbreaks, prediction, planning and control-oriented interventions are at the core of a particular style of development. We see this in many areas, from efforts to address finance and poverty, to those tackling environmental and climate change. Models of various sorts are central to these approaches, as are advice networks that tend to be narrow and technocratic. The systems of administration and intervention that follow are top-down and oriented towards blueprint plans, all far removed from people’s lives and practices and the uncertainties arising as a result. Where uncertainties are admitted these are reduced to the domain of calculable risks, where probabilities can be assigned, as in many early warning, disaster risk reduction and contingency planning systems. Under-acknowledged are the uncertainties (where probabilities of potential outcomes are not known), ambiguities (where outcomes are contested among different groups) and forms of ignorance (where neither outcomes nor probabilities can be assigned) that prevail in the complex world of development decision-making and field practice (Stirling, 2010). Ignoring these in fallacious attempts at control opens up error and danger (Stirling and Scoones, 2020).

If uncertainties are to be embraced more fully in development – from disease control to climate change, social protection and economic planning – then a more plural, conditional approach to science and expertise is required. The standard risk-oriented predict, plan and control approaches of modernist development are found seriously wanting. And with these, so too are Northern-dominated cultures and institutions of expertise. This means a different approach to modelling, encompassing a diversity of inputs and sources of knowledge, rooted in a deliberative and inclusive approach to science advice.

COVID-19 has also shown brutally that reliance on narrow sources of evidence and expertise, and rigid plans, can be highly problematic. Instead, to foster reliability in the face of complexity, turbulence and ‘mess’ – i.e. the real world – a new approach is required (Roe, 2013). In ‘critical infrastructures’ – whether energy, water, nuclear power stations or air traffic control – reliability emerges through tracking of diverse scenarios (including through modelling) combined with real-time attention to system functioning on the ground (derived from front-line professionals and those in the field). These fields of evidence are connected by ‘reliability professionals’ who can link and communicate between and across networks to ensure reliability (Roe, 2013). This is as applicable to the critical infrastructures of health systems as it is to food or economic systems, but is often missed due to the disconnected foci on broad systems modelling and local knowledges and practices.

A danger with COVID-19 is that its emergence is seen as unprecedented, and that it is argued that improved models could have predicted and stopped it and strong enforcement of ‘top-down’ public health measures could have controlled the escalation – in turn reinforcing this kind of approach to shocks more generally. But lessons from multiple disease outbreaks and development initiatives suggest that uncertainty is always present and acknowledging it, in conjunction with generating reliability, must be at the core of development policy and practice. This will require a fundamental rethinking of how expertise of multiple sorts and new forms of professionalism are convened and combined. Now, more than ever, project and programme styles that emphasise learning, iterative adaptation, flexible action and equitable relationships amongst diverse actors (Chambers, 2017) need to move from the margins to centre-stage.

### 3.2 Resilient economies

In order to contain COVID-19, governments around the world have shut down economies on an unprecedented scale. This has had a huge impact on employment and economic output, with estimates that over US$5 trillion will be wiped off the world economy, the equivalent of the whole of Japan’s economy.^3^ The scale of economic impact has taken many by surprise, but in modelling of potential impacts of a global outbreak of avian influenza, spreading rapidly between humans, including national border closures and global and local movement restrictions, the World Bank predicted long ago that the impact could be up to 4.8 percent of global GDP in the first year (Burns et al., 2006). Subsequent pandemic preparedness planning at the global level often referred to this work, but this did not seem to have much effect; the numbers were too large, threats too distant and impacts too unimaginable.

Central banks had developed contingency plans for major shocks, including pandemics, which consistently appear at the top of risk registers, and had used liberal ‘quantitative easing’ following the financial crisis of 2008. But the scale of the COVID-19 economic impact – both in terms of loss of economic activity and the requirements for credit and loan finance to keep businesses functioning – has been beyond any earlier estimates, requiring some major rethinks (Tooze, 2020). In the absence of economic contingency plans for a pandemic on this scale, national governments around the world have been scrambling to make up economic policy on the spot, rapidly jettisoning long-established rules about government spending limits, debt caps and fiscal austerity. In some countries, substantial income support for those who have lost livelihoods has been offered, but this is far from universal, with many people left to survive unaided. With the sudden decline in business activity, this has demonstrated how precarious work in the modern economy is.

Lessons from earlier disease outbreaks are of limited relevance to addressing COVID-19s impacts on the global economy. The Spanish influenza of 1918 caused major economic destruction globally, but on the back of a four-year war, and in economies that were far less connected, and much smaller than today (Barry, 2005). Periods of war transform economies, but often this is through shifts to new activities, as workers are redeployed to war-time industries or are recruited into armies, and post-war booms are common, as economies bounce back. More recent disease episodes have not caused such widespread economic collapse. Another coronavirus disease, SARS, spread globally in 2003, causing significant economic damage, as countries implemented movement restrictions and quarantining. However, the overall cost was not as great as predicted at the time, and economies returned to previous levels of economic activity relatively quickly, as connections with the still-functioning global economy were re-established (Keogh-Brown and Smith, 2008). Today, the situation is dramatically different, as all economies across the world are affected, although not all in exactly the same way and at exactly the same time.

Disasters of course may open up opportunities for some, as investors buy up weakened industries and speculate on investments when prices are low. Such ‘disaster capitalism’ (Klein, 2007) has more reach when some areas are affected – say by an earthquake – and others can move in to exploit the situation. Some are capitalising on local shortages or providing new on-line and delivery services (e.g. Amazon in many countries), acting to restructure economies. Only once the crisis subsides can we evaluate how assets have been shifted, new speculative investments made and business concentration and monopolies extended or fragmented (Davis, 2020).

Recent experiences with the COVID-19 lockdowns have shown clearly how labour is central to economies and how much of this so-called ‘essential work’ is low-paid, precarious and carried out by women, ethnic minorities and migrants (Meadway, 2020). When jobs disappear at a stroke, it is frequently the rural areas where support is sought, highlighting how crucial, even in an urban-dominated, globalised economy, rural connections are for social reproduction and sustaining the wider economy. Yet rural labourers have also been some of those to suffer most from COVID-19 and policies, due to movement restrictions and health risks from working in unsanitary conditions, threatening both their livelihoods and wider economic and food system sustainability (IPES-Food, 2020).

Much policy discussion has centred on the need for massive stimulus packages to get the economy moving after the pandemic crisis recedes. The aim is to encourage growth and get economies ‘back on track’ through forms of Keynesian-type stimulus, as used after the Second World War. Lessons – both positive and negative – are also drawn from recovery attempts after the 2008 financial crisis, based on a major re-capitalisation of the global banking system (Tooze, 2020). However, these approaches focused on fixing existing structures through standardised investments to boost economic growth miss opportunities for a deeper rethinking of economic systems for a post-pandemic transformation.

For what COVID-19 has exposed is the fragility of the current globalised capitalist economy, with its reliance on *trans*-continental financialised transactions, just-in-time production and long, carbon-consuming international supply chains. It has also shown how local economies are frequently more resilient to massive shocks and can provision effectively and efficiently during a crisis. Embedded, inclusive, often informal and unruly, economies, rooted in mutualism and solidarity, have flourished. For example, the area of food provisioning has seen a remarkable upsurge of solidarity and grassroots activism, from widespread donation of food to the destitute in India and Pakistan, to the provision of mobile meals to disadvantaged populations in the US and Canada. Communities have come together to plug gaps in the system and help those in need, with civil society groups sometimes working together with supportive state actors. For example, in the highly decentralised Indian system, the state of Kerala has led the way in its response to COVID-19, by ensuring food distribution via free community kitchens run by women's networks (IPES-Food, 2020). Such examples are reminiscent of community solidarities that have emerged and enabled resilience in earlier disasters (Solnit, 2009). The question is: are such solidarities confined to the particular context of an emergency, or do they offer glimpses of alternative economies for the future?

This of course links to wider debates that have a long pedigree in development studies. Early considerations of the ‘informal economy’ (Hart, 1973) highlighted how such activities provide wide opportunities, and can foster local development, even without ‘up-grading’ and formalisation. Denigrated as backward, unregulated and not contributing to formal economic activity, the informal economy is seen as in need of transformation by policymakers (Meagher, 2013). Cooperatives and worker-owned industries were popularised in the 1970s, such as the ‘Lucas Plan’ (Smith et al., 2016), but again were seen as marginal to mainstream industrial growth and global value chain development. Yet, as Thorpe and Gaventa (2020) show, through a study of 28 cases from across the world, local economic activity can be profitable, inclusive and sustainable under the right conditions. Key features of effective economic governance identified include distributed authority in the management of a business; engagement and mobilisation of workers and others to effect change; networks and coalitions as central to economic activity; deliberation for distributed decision-making and democratised knowledge for collective action.

Such features are emphasised in economic responses to COVID-19 and other major shocks, suggesting possibilities for more resilient, post-capitalist alternatives. A key theme is the importance of the commons – shared resources managed collectively – in providing economic activity and livelihoods, and the need for mutuality and solidarity to support those involved, across supply chains and between producers and consumers, as part of joint, collective economic action (Bollier and Helfrich, 2014). Such approaches recall older styles of economic organisation – from shared rights to resources in forests in Africa to mutual societies in support of workers in early industrial England, for instance, each rooted in common property arrangements and collective management, with markets embedded in society (cf. Polanyi, 2001 [1944]). Such examples show how effective, sustained economic activity can be supported through shared networks, not individualised, competitive economic activity, as in the conventional economic models of development textbooks. As COVID-19 has shown, such styles of economic activity can persist through crises, in ways that other forms of economic organisation simply cannot.

The COVID-19 experience has also highlighted wider ideas of value in the economy – of ‘essential workers’; of local production and markets; of networks of sharing at the community level and of low-carbon, sustainable sourcing of goods. Rethinking ‘value’ and ‘purpose’ in the economy (Mazzucato, 2018) requires deliberating on the directions of economic change. Is ‘growth’ necessarily the sole objective, or are there other values, such as equity or sustainability that are important for human well-being? A focus on social reproduction suggests an emphasis on ‘life-making’ not ‘profit-making’ (Jaffe, 2020), highlighting the contradictions between care and capital (Fraser, 2016). Rather than stimulus packages returning to the *status quo* of a high-carbon economy, measured in terms of narrow indicators of GDP growth, diverse alternatives are suggested (D’Alessandro et al., 2020). This raises questions of how societally-governed ‘limits’ are negotiated in social and economic life (Kallis, 2019) and how social floors to protect those in need – including mechanisms such as Universal Basic Income – can be combined with approaches that ensure that economies function sustainably, within ‘planetary boundaries’. Principles of collaboration, regeneration and circularity, rather than extraction and growth, thus become the defining guides for economic development (Raworth, 2017).

Through thinking about how economies become resilient in the face of recurrent shocks, relations between states and markets are challenged too. Rather than states being shrunk to allow markets to function unfettered, as in the neoliberal era, or as sources of funding to prop up banks or ailing industries, as in post-crisis moments, ‘post-capitalist’ states can take on new functions to improve systemic resilience, including public ownership of vital services and support for basic incomes (Mason, 2016, Bello, 2008). An ‘entrepreneurial state’ can foster innovation, encouraging long-term, patient finance (Mazzucato, 2013) supporting particular directions of sustainable economic development. Progressive regulatory environments for capital can in turn steer transformations towards more sustainable, equitable futures through ‘just’ and ‘green’ transformations (Newell, 2015). Equally, radical uncertainties are a feature of our complex, connected world, so the design of institutions, businesses and infrastructures – and indeed the wider economy – for reliability, with built-in redundancy and adaptive capacities, are essential (Roe and Schulman, 2008). In sum, expanding the criteria for ‘success’ in economic development beyond growth and profit to thinking about equity, resilience and sustainability suggests a very different set of directions for economic development, requiring diverse, new skills.

A particular type of neoclassical economics has dominated development studies for decades, rooted in individualised behaviour, linked to market efficiency, business profit and aggregate economic growth, and committed to a particular style of capitalist development. While debates about the relative role of states and markets have occurred over time and been repeatedly revisited (Colclough and Manor, 1993, Leach, 2016), objectives of economic growth as development have remained, even if tweaked in favour of greater equality, more sustainability and so on. The radical rupture that COVID-19 has created means that old assumptions have been challenged, and a more fundamental rethink of capitalism and development is needed. This requires a transformative economics for sustainable, inclusive development that can survive future shocks, whether pandemics, climate change, financial instability or conflict, which is rooted in ideas of inclusive, solidarity economies (Utting, 2015), where long-held notions of modernist ‘progress’ are rethought and sustainability and equity are central.

### 3.3 Re-configuring citizen-state relations

The public health measures required during a significant disease outbreak necessarily shift state-citizen relations, and these changed relationships will be the driver of transformations to follow. Forcing lockdowns, restricting movements, requiring new behaviours and creating surveillance to monitor populations, all involve the exercise of state power and implementation and depend on trust between citizens and authorities and in sources of expertise. States of emergency have historically been used to extend power and abuse rights in the longer term; and today there is evidence some leaders are using COVID-19 to do just this (Smith and Cheeseman, 2020). There is also evidence that untrusting publics have been pushed to their limits, provoked into unruliness, as in Malawi when people revolted against lockdown orders and the authority of a government already perceived to be illegitimate. In a further twist, human rights coalitions took the government to court and won, resulting in the suspension of restrictions (Dodsworth and Cheeseman, 2020). The control of COVID-19 and the emergence of exit strategies are a massive test of authority and accountability, and the need to be inclusive of all citizens.

Trust is both a measure of state-citizen relations and an enabler of effective response to diseases and development more generally. Experience during the West African Ebola outbreak showed how critical trust was. Trust and any sense of a social contract was lacking because of the legacy of slavery, colonialism, war and the failure of state provisioning in previous decades. Communities had neither trust in the health system, their government or outsiders; and nor were they trusted by these authorities and outsiders to know what was best for them. Building trust required tangible improvements to services, but also dialogue and relationship building. It was only when communities, supported by truly representative leaders, were able to take the lead that critical behaviours began to change – such as around safe burial and quarantining (Wilkinson et al., 2017), shifting the course of the epidemic.

Trust between authorities, medical establishments and citizens is often lacking at the onset of epidemics but can be built as part of a response and re-shaped in the process. In the early phases of the HIV/AIDS pandemic, public health efforts were upset by blame and stigmatisation. AIDS was seen as a disease of ‘gays’, ‘foreigners’, ‘sex workers’, ‘truck drivers’ for instance, and not a responsibility of all citizens (Parker, 2002). In some cases, states ignored mainstream medical advice, failed to develop national programmes and calls for universal access to emerging antiretroviral therapies. This was the case in South Africa, where support of ‘AIDS dissidents’ who peddled an alternative understanding of the science was rooted also in perceptions on the part of the president and health minister that prevailing narratives of the origins of HIV in Africa denigrated African sexuality and lifestyles, and undermined a positive vision of a new African renaissance. The resultant lack of political will in ‘rolling out’ free antiretrovirals had marked consequences for those infected by the virus in South Africa, which were those most marginalised by poor access to healthcare and the structural violence of the Apartheid period. Only when wider mobilisations – such as through the Treatment Action Campaign – occurred, did new relationships between citizen initiatives and state investment become realised which led to court action and finally the initiation of a free anti-retroviral therapy programme in the state health sector. Scepticism that people living in precarity would not be able to maintain adherence to antiretrovirals or be engaged in self-management and health education initiatives was dealt a blow by civil society initiatives built on principles of patient empowerment, collective action and holding the state to account to uphold the right to health and to other social protection measures (MacGregor, 2010). Across Africa, a surge of community-based organisations was partly linked to increases in global health funding, and provided homebased care as well as support for those affected by HIV led to initiatives in income generation and for orphans and vulnerable children (Edström and MacGregor, 2010).

These examples show how important state-citizen alliances are in times of crisis, and beyond. This is evident again with the flourishing of ‘mutual aid’ initiatives during COVID-19, supported by local and national states, as well as diaspora networks, religious organisations, business networks, philanthropists and others. Trust, inclusive collaboration, collective action and mutuality are the watchwords, complemented by ethics of care, respect and empathy. And here there are links to inequality: trust is lower in more unequal societies, and low trust and high inequality have been shown to hinder cooperation, collective action, inclusive politics and economic development (Justino, 2015). This points to the potential for a new style of politics, embedded in communities and egalitarian norms, yet supported by a trusted, accountable state.

The ‘bottom-up’ COVID-19 responses have been impressive globally, emerging through force of circumstance. But can such experiences be translated into longer-term struggles for transformation, linking structural, systemic and enabling change (Scoones et al., 2020)? Beyond addressing the pandemic there remain many other development challenges that require such a new style of politics, not least perhaps the largest one of all: that of transformations towards a low-carbon sustainable future. In our discussions of the politics of ‘green transformations’ more broadly (Scoones et al., 2015), we specifically identified the need for alliance-building, across technology-led, business-led, state-led and citizen-led transformations. Looking to earlier successful transformations where radical change has been effected, it is always connections between actors through networks that help forge an alternative vision. This inevitably requires hard political negotiations, across unequal power gradients. Alliances for sustainability require political choices and the challenging of incumbent interests, reliant for example on deeply-entrenched fossil fuel capitalism. Yet, changes are possible and may happen quite rapidly as new progressive narratives for change gain a foothold, new technologies emerge, alternative networks form and political interests realign.

Crises can open up new forms of relationship and action, and can demonstrate alternatives, whether these are new styles of local politics and state-citizen relationship that can assist in an immediate emergency and persist beyond it, reformed approaches to global coordination or more fundamental transformations in economy and society towards new pathways of development. But ruptures can also close down, reinforcing the *status quo* and shoring up incumbent power. COVID-19, for example, offers opportunities for the spread of surveillance-led authoritarianism, as citizens accede to control of their data and lives in the name of public health; states may act to shore up big business in the name of protecting jobs and the economy, rather than seeking radical transformations and the rhetoric of nationalist, authoritarian populisms frequently gains a hearing in times of crisis, as leaders claim they can protect people from external threats (Rodrik, 2018).

What the future will hold remains uncertain, but major challenges, such as COVID-19, do both expose fractures and contradictions and offer opportunities for change, which ultimately will depend on political choice and mobilisation. This must address both the structural dimensions, challenging incumbent power, while accepting that change is not linear, and must embrace uncertainty, complexity and unruliness in politics, as in economy, ecology and society – vital to forging and moving forward with a politics not of authoritarianism but of solidarity and care.

## 4 Conclusion

In surveying the recent history of zoonotic diseases, COVID-19 is not unexpected. As we have outlined, there have been lessons and warnings for some time. The issues around the origins, unfolding and impacts of epidemics that we have highlighted – political economies, ecologies and intersecting inequalities, both structural and unruly – have come into sharp relief through the COVID-19 experience, but were central to our explorations of past disease outbreaks, from HIV/AIDS to avian and swine influenza to Ebola and Zika. Together, they suggest the need for a major rethinking not only of pandemic preparedness and response, but also of development theory and practice, whether around the role of science, evidence and expertise; around economies and the nature of ‘value’ and as part of exploring the politics of state-citizen relationships in transformations more broadly.

Our analysis thus reveals the need for development approaches that can anticipate and respond to future, uncertain shocks – whether pandemics, climate change, financial turbulence or something else we have not even thought of. This means both revealing and challenging the structural conditions, power relations and political economic orders that create risks and vulnerabilities in the first place, while also accepting the need for flexible, contingent and negotiated responses in the face of uncertainty and context-specific complexity. Lasting transformations must address fundamental matters of power and politics, including challenging incumbent institutions and interests, at the same time as fostering hopeful, innovative alternatives (Solnit, 2009, Klein, 2007). Post-pandemic transformation also means embracing uncertainty and fostering often unruly, diverse alternatives that allow economic, social and political systems to transform towards more equitable and sustainable development pathways. It means rejecting the illusions of ‘control’, whether via technology, the market or state intervention, and enabling a more caring, inclusive, convivial approach to development (Scoones and Stirling, 2020); one in which knowledge and learning from diverse people and places have key roles to play and are harnessed to complement formal institutional measures.

These lessons centre on the need to embrace fundamental, transformative change, to navigate uncertainty and prepare for turbulence as a central requirement of development, North and South. For these are universal challenges, precipitated by shocks and stresses that have global reach, whether disease pandemics, climate change or the reverberations of economic volatility through an interconnected globalised economy. The conventional modalities of development, crafted in the period after 1945, have emphasised a control-oriented approach, premised on modernist visions of prediction and planning. Whether under state-led capitalism or free-market neoliberalism, the assumptions of such a development model have been challenged by the turbulent and complex effects of pandemic disease, and the dislocations in economies and societies that this produces.

The rethinking needed extends to how we conceive of ‘development’ itself, and its geographies and power relations. This must not be the preserve of those intervening in the global South – of projects and programmes delivered through aid flows – but a much more universal concern. For the COVID-19 humanitarian health and development crisis, and the inequalities and precarities that this has exposed, has been felt as much in New York as it has in Nairobi. Questions of social protection, basic income for sustainable livelihoods, supporting informal economies, as well as universal healthcare provision, are today being posed across the world, and not just as an ‘othered’ form of ‘development’, only relevant somewhere else. The ‘universality’ endorsed and promised in the UN Global Goals, and signed up by and applicable to all countries across the world, yet in practice embraced weakly in Western domestic policy agendas, may finally have come of age. It should now be embraced fully. With this, we may hope for a further deconstruction of the colonial assumptions and power relations that have long beset development studies and practice, and strengthening of a ‘decolonised’ agenda, grounded in more equitable sharing of knowledge and resources, supported by continuous challenging of historically-embedded power dynamics.

Through exploring the experience of COVID-19 to date and reflecting on past epidemics, we have highlighted areas where an opening up of debate is required, often suggesting new ways of thinking and acting that push the boundaries of development studies and practice. While recognising the failures to learn lessons from past disease outbreaks where similar themes have emerged, we strike a note of optimism. The scale and depth of the COVID-19 crisis, and its North-South universality, perhaps mean that, this time, progressive transformations will emerge – in different places, in different ways – that embrace uncertainty, unruliness and inevitable complexity, while equally confronting the structures of mainstream capitalist development that give rise to persistent crises, generate unequal vulnerabilities and impede progressive change. Of course, power and politics will intervene, incumbent interests will naturally resist and opportunists may fill the vacuum, but the required changes are in the end political choices, requiring democratic struggle and mobilisation. If such far-reaching transformative change does not emerge, the project of ‘development’ will have failed, and future shocks – for they will surely come – will wreak even greater havoc.

## Declaration of Competing Interest

The authors declare that they have no known competing financial interests or personal relationships that could have appeared to influence the work reported in this paper.

## Footnotes

2 See, https://steps-centre.org/covid-19-coronavirus-resources-research-epidemics-pandemics/.

3 https://www.bloomberg.com/news/articles/2020–04-08/world-economy-faces-5-trillion-hit-that-is-like-losing-japan.
